# Supplementary material for: Effect of a Second Pregnancy on the HPV Serology in Mothers Followed Up in the Finnish Family HPV Study
Source: Viruses. 2023 Oct 18;15(10):2109. doi: 10.3390/v15102109 (PMC10612095; doi:10.3390/v15102109)
Supplement: Supplementary file 1 [file viruses-15-02109-s001.zip › viruses-2630990-Supplementary.pdf]

Spplementary Table S1. HPV6, HPV11, HPV16, HPV18 and HPV45 serostatus (MFI >200 and MFI >400) during the follow-up, stratified by the 2nd pregnancy. Significant differences between groups are bolded.

	**2nd pregnancy**		**Baseline** MFI > 200	**Baseline** MFI > 400	**12 mo** MFI > 200	**12 mo** MFI > 400	**24 mo** MFI > 200	**24 mo** MFI > 400	**36 mo** MFI > 200	**36 mo** MFI > 400
			n (%)		n (%)		n (%)		n (%)	
HPV6	Yes	Seropositive	**40 (44.9)** [a]	29 (32.6)	56 (62.9)	34 (38.2)	55 (63.2)	37 (42.5)	44 (49.4)	37 (41.6)
		Seronegative	**49 (55.1)** [a]	60 (67.4)	33 (37.1)	55 (61.8)	32 (36.8)	50 (57.5)	45 (50.6)	52 (58.4)
	No	Seropositive	**139 (58.4)** [a]	97 (40.8)	140 (71.8)	97 (49.7)	123 (70.3)	79 (45.1)	96 (55.8)	63 (36.6)
		Seronegative	**99 (41.6)** [a]	141 (59.2)	55 (28.2)	98 (50.3)	52 (29.7)	96 (54.9)	76 (44.2)	109 (63.4)
HPV11	Yes	Seropositive	13 (14.6)	**6 (6.7)** [b]	21 (23.6)	9 (10.1)	19 (21.8)	8 (9.2)	12 (13.5)	5 (5.6)
		Seronegative	76 (85.4)	**83 (93.3)** [b]	68 (76.4)	80 (89.9)	68 (78.2)	79 (90.8)	77 (86.5)	84 (94.4)
	No	Seropositive	57 (23.9)	**36 (15.1)** [b]	57 (29.2)	31 (15.9)	43 (24.6)	26 (14.9)	27 (15.7)	12 (7.0)
		Seronegative	181 (76.1)	**202 (84.9)** [b]	138 (70.8)	164 (84.1)	132 (75.4)	149 (85.1)	145 (84.3)	160 (93.0)
HPV16	Yes	Seropositive	28 (31.5)	20 (22.5)	37 (41.6)	25 (28.1)	31 (35.6)	22 (25.3)	25 (28.1)	12 (13.5)
		Seronegative	61 (68.5)	69 (77.5)	52 (58.4)	64 (71.9)	56 (64.4)	65 (74.7)	64 (71.9)	77 (86.5)
	No	Seropositive	81 (34.0)	46 (19.3)	79 (40.5)	48 (24.6)	57 (32.6)	37 (21.1)	49 (28.5)	26 (15.1)
		Seronegative	157 (66.0)	192 (80.7)	116 (59.5)	147 (75.4)	118 (67.4)	138 (78.9)	123 (71.5)	146 (84.9)
HPV18	Yes	Seropositive	**10 (11.2)** [c]	3 (3.4)	**13 (14.6)** [d]	8 (9.0)	16 (18.4)	8 (9.2)	**14 (15.7)** [e]	8 (9.0)
		Seronegative	**79 (88.8)** [c]	86 (96.6)	**76 (85.4)** [d]	81 (91.0)	71 (81.6)	79 (90.8)	**75 (84.3)** [e]	81 (91.0)
	No	Seropositive	**56 (23.5)** [c]	23 (9.7)	**62 (31.8)** [d]	27 (13.8)	43 (24.6)	25 (14.3)	**47 (27.3)** [e]	27 (15.7)
		Seronegative	**182 (76.5)** [c]	215 (90.3)	**133 (68.2)** [d]	168 (86.2)	132 (75.4)	150 (85.7)	**125 (72.7)** [e]	145 (84.3)
HPV45	Yes	Seropositive	5 (5.6)	1 (1.1)	8 (9.0)	4 (4.5)	9 (10.3)	3 (6.3)	6 (6.7)	2 (2.2)
		Seronegative	84 (94.4)	88 (98.9)	81 (91.0)	85 (95.5)	78 (89.7)	84 (96.6)	83 (93.3)	87 (97.8)
	No	Seropositive	26 (10.9)	9 (3.8)	24 (12.3)	11 (5.6)	15 (8.6)	11 (6.3)	14 (8.1)	5 (2.9)
		Seronegative	212 (89.1)	229 (96.2)	171 (87.7)	184 (94.4)	160 (91.4)	164 (93.7)	158 (91.9)	167 (97.1)

p-values = [a] 0.034, [b] 0.043, [c] 0.013, [d] 0.002, [e] 0.044

Abbreviations: MFI = mean fluorescence intensity, mo = months

Cut-off value for seropositivity was MFI > 200 or MFI > 400 (stringent)
